# Establishment of a stress granule reporter system for evaluating *in vitro* colon toxicity

**DOI:** 10.1080/19768354.2024.2364673

**Published:** 2024-06-17

**Authors:** Namjoon Cho, Da-Min Jung, Eun-Mi Kim, Kee K. Kim

**Affiliations:** a Department of Biochemistry, College of Natural Sciences, Chungnam National University, Daejeon, Republic of Korea; b Department of Bio and Environmental Technology, College of Natural Science, Seoul Women’s University, Seoul, Republic of Korea

**Keywords:** Colon toxicity, HCT116, stress granules, G3BP1, real-time monitoring

## Abstract

Exposure to toxic molecules from food or oral medications induces toxicity in colon cells that cause various human diseases; however, *in vitro* monitoring systems for colon cell toxicity are not well established. Stress granules are nonmembranous foci that form in cells exposed to cellular stress. When cells sense toxic environments, they acutely and systemically promote stress granule formation, with Ras GTPase-activating protein-binding protein 1 (G3BP1) acting as a core component to protect their mRNA from abnormal degradation. Here, we knocked in green fluorescent protein (GFP)-coding sequences into the C-terminal region of the *G3BP1* gene in a human colon cell line through CRISPR-Cas9-mediated homologous recombination and confirmed the formation of stress granules with the G3BP1-GFP protein in these cells under cellular stress exposure. We demonstrated the formation and dissociation of stress granules in G3BP1-GFP expressing colon cells through real-time monitoring using a fluorescence microscope. Furthermore, we validated the toxicity monitoring system in the established colon cell line by observing stress granule formation following exposure to dihydrocapsaicin, bisphenol A, and sorbitol. Taken together, we established a stress granule reporter system in a colon cell line, providing a novel assessment for the real-time monitoring of colon toxicity in response to various chemicals.

## Introduction

The colon is the last part of the digestive system and absorbs water and nutrients from food. Moreover, the colon is a site for the fermentation of indigested matter *via* colonic bacteria. The microbiome produces various metabolites and nutrients that are pivotal to health (Krautkramer et al. 2021; Lee et al. 2023). Because of the numerous microbiomes and molecules, colon cells are constantly exposed to cellular toxicity; therefore, intestinal stem cells grow rapidly and regenerate intestinal tissues in the human body (van der Flier and Clevers 2009). Oral administration is one of the most common and preferred approaches to drug delivery in humans (Zhang et al. 2023). Oral medications are absorbed by the digestive tract, including the colon; therefore, they may have adverse effects on colon cells. Repeated exposure of colon cells to toxic molecules can be a risk factor for various human diseases, including inflammatory bowel disease and colon cancer (Caruso et al. 2020; Gulhane et al. 2016; Laugerette et al. 2012; Sepich-Poore et al. 2021). Notably, the consumption of instant and industrially manufactured foods containing additives has increased worldwide. Therefore, a system that can accurately and extensively monitor the cytotoxicity of these chemicals in colon cells is required.

When human cells encounter cellular stress, they activate defense mechanisms to protect against cellular damage. One such pathway is the stress granule pathway (Anderson and Kedersha 2002a). Stress granules are nonmembranous organelles that are rapidly formed by cellular stress to suppress global translation and prevent the degradation of mRNAs (Marcelo et al. 2021). HRI (Activated by oxidative stress), PERK (Activated by ER stress and unfolded protein response), PKR (Activated by viral infection and intracellular double-stranded RNA level), and GCN2 (Activated by amino acids starvation and UV irradiation) are each activated by a variety of different cellular stress stimuli and then they promote the phosphorylation of the α subunit of eukaryotic initiation factor 2 (eIF2α) at serine 51 (Anderson and Kedersha 2002b, 2006, 2009; Donnelly et al. 2013; Jung et al. 2023; Kedersha et al. 2002, 1999; Krishna and Kumar 2018). Phosphorylated eIF2α represses the translation and dissociates the translation initiation complexes from mRNAs (Kedersha et al. 2002; Protter and Parker 2016). As a result, G3BP1 acts as a scaffold and seed protein for liquid–liquid phase separation, leading to the aggregation of untranslated mRNAs, RNA-binding proteins, translation factors, and other proteins, ultimately forming stress granules (Campos-Melo et al. 2021; Choi et al. 2019; Park et al. 2017; Protter and Parker 2016).

In this study, we aimed to establish an *in vitro* system for monitoring toxicity in colon cells using a stress granule mechanism. Stress granules are ubiquitously formed in eukaryotic cells in response to several cellular stressors (Choi, Choi, et al. 2022; Choi, Kim, et al. 2022; Tweedie and Nissan 2021; Valiente-Echeverria et al. 2012). Moreover, stress granule formation is mediated by phase separation; therefore, this reaction is faster than other stress response reactions that mediate transcription or translation efficiency (Hofmann et al. 2021). Finally, G3BP1, the core component of stress granules, is continuously expressed in most human cells and is ready for use in the formation of SGs (Aulas et al. 2015; Yang et al. 2020). Therefore, we generated a stress granule reporter colon cell line expressing G3BP1 fused with GFP (G3BP1-GFP), which will serve as a valuable system for sensitively reporting colon cell toxicity in response to a broad range of cellular stress stimuli and for monitoring real-time responses to cellular conditions upon exposure to chemicals.

## Materials and methods

### Cell culture

HCT116 human colon cancer cells were obtained from the American Type Culture Collection (ATCC) and cultured in Dulbecco's Modified Eagle's medium (DMEM; WELGENE, LM001-05) supplemented with 10% fetal bovine serum (Gibco, 12483-020) and 1% penicillin–streptomycin (WELGENE, LS202-02). Cells were maintained at 37°C CO_2_ incubator in a humidified atmosphere.

### Construction of plasmid DNAs

The optimized sgRNA sequences targeting the 3′ terminal region of G3BP1 coding sequences were designed using the CRISPOR program (http://crispor.tefor.net/) as follows: 5′-CGA CGA GAT AAT CGC CTT CG-3′ (top) and 5′-AAA CCG AAG GCG ATT ATC TC-3′ (Bottom). The primers were annealed using a PCR machine and cloned into a px330 vector. To construct a DNA donor plasmid containing GFP-coding sequences, the homology arms were cloned into the pDsRed-Express2-1 vector. Primers used for the DNA donor construction are as follows: 5′ homology arm amplifying 5′-ATA TAG ATC TCA ATG GCG TGA TCT TGG CT-3′ (Bgl2) and 5′-CTG TCG TGG CGC AAG C-3′; GFP coding sequence (CDS) amplifying 5′-**AAG GGG GCT TGC GCC ACG ACA G**AT GGT GAG CAA GGG CGA-3′ and 5′-**GCA TGA AGA TCC ATG AAG AT**T TAC TTG TAC AGC TCG TCC ATG-3′; 3′ homology arm amplifying 5′-ATC TTC ATG GAT CTT CAT GCA G-3′ and 5′-TAG CGG CCG CAG GCA CAA CAG TTT TGC TC-3′ (Not1). Underlined and bold nucleotides represent restriction enzyme sites and additional complementary sequences for overlap extension PCR, respectively.

### Establishment of G3BP1-GFP knock-in cells

HCT116 cells were seeded in the 6-well plate (SPL, 30006; 3 × 10^5^ cells/well) for 24 h. Subsequently, 0.5 μg of DNA donor plasmid and 0.5 μg of px330 plasmid containing G3BP1 target sgRNA were transfected using PolyMag reagent (Chemicell, 9003), according to the manufacturer’s instructions. After transfection for 7 days, the cells were trypsinized, and GFP-positive cells were sorted and seeded at one cell per well in 96-well plates (SPL, 30096).

### Immunoblot analysis

Cells were seeded in the 6-well plate (3 × 10^5^ cells/well) for 24 h, and lysates were obtained using RIPA lysis buffer (Thermo Fisher Scientific, 89900). Protein concentrations were quantified using a BCA assay (iNtRON Biotechnology, 21071) and denatured by adding Laemmli sample buffer (Bio-Rad, 1610737) containing 5% β-mercaptoethanol (Sigma-Aldrich, T9281). Prepared protein lysates were separated using the SDS-PAGE gel, transferred to the nitrocellulose membrane (Merck, HATF00010), and then blocked the membrane using 5% non-fat dry milk (ROCKLAND, B501-0500) dissolved in phosphate-buffered saline containing 0.05% Tween-20 for 1 h at 25°C. The primary antibodies were diluted in 5% non-fat dry milk and treated to the blocked membrane overnight at 4°C. After washing with primary antibodies, horseradish peroxidase-conjugated secondary antibodies were diluted in 5% non-fat dry milk and incubated for 1 h at 25°C, followed by washing of the membrane. Protein bands were detected using a WSE-6200H LuminoGraph II (ATTO). The primary antibodies are as follows: anti-G3BP1 (Santa Cruz Biotechnology, sc-365338; 1:500), anti-GFP (Santa Cruz Biotechnology, sc-9996; 1:500), anti-GAPDH (Meridian Life Science; 1:5000), anti-phospho-eIF2α (Cell signaling, 3597 L; 1:1000), anti-eIF2α (Santa Cruz Biotechnology, sc-133132; 1:1000), and anti-Tubulin (Sigma-Aldrich, T5168; 1:5000) antibodies.

### Genomic DNA extraction and PCR

Genomic DNA was extracted from 1 × 10^6^ cells using a G-spin^TM^ Total DNA Extraction Kit (iNtRON Biotechnology, 17045) according to the manufacturer’s instructions. PCR was performed using a hot-start PCR master mix (Bioneer, K-2630), and the products were subjected to agarose gel electrophoresis. The primers confirming the knock-in of GFP into the *G3BP1* gene are as follows: 5′-CCA TGG AGA AGG CTG GGG-3′ and 5′-CAA AGT TGT CAT GGA TGA CC-3′.

### Immunofluorescence and live cell imaging

HCT116 cells were seeded in the 4-well chamber slide (Nunc, 154526; 3 × 10^4^ cells/well) for 24 h, and then fixed using 4% paraformaldehyde for 10 min at 25°C, followed by permeabilized using 0.5% Triton X-100 in phosphate-buffered saline for 15 min at 25°C. Cells were blocked using the blocking buffer (5% goat serum, 1% bovine serum albumin, and 0.05% Tween 20 in phosphate-buffered saline) for 1 h at 25°C. The anti-GFP (Santa Cruz Biotechnology, sc-9996; 1:250) and anti-G3BP1 (Santa Cruz Biotechnology, sc-98561; 1:500) antibodies were diluted in the blocking buffer and treated to cells for overnight at 4°C. After washing the primary antibodies, Goat anti-mouse IgG antibody conjugated to Alexa Fluor 488 (Thermo Fisher Scientific, A11008; 1:500), goat anti-rabbit IgG antibody conjugated to Alexa Fluor 594 (Thermo Fisher Scientific, A11012; 1:500), and 4ʹ,6-diamidino-2-phenylindole (DAPI; Thermo Fischer Scientific, D1306; 1:1,000) were diluted in the blocking buffer and treated for 1 h at 25°C, followed by washing and mounting with ProLong^TM^ Gold antifade reagent (Thermo Fischer Scientific, P36930). To assess live imaging, cells were seeded in μ-Slide 4 well chamber slide (ibidi, 80426; 3 × 10^4^ cells/well) for 24 h, followed by GFP detecting under 37°C in a humidified 5% CO_2_ atmosphere. Cells were treated with dihydrocapsaicin (Sigma-Aldrich, M1022), bisphenol A (Sigma-Aldrich, 239658), and sorbitol (Sigma-Aldrich, 56755) for live cell imaging analysis. PERK inhibitor (GSK2606414; Sigma-Aldrich, 516535) were treated for stress granule recovery analysis. All images were captured using a Zeiss LSM 880 confocal laser scanning microscope (Carl Zeiss).

### Cell viability analysis

Cells were seeded in the 96-well plate (5,000 cells/well), incubated for 48 h, and treated the chemicals. To assess cell viability, 20 microliter of MTS solution (Promega, G3581) was added to each well and incubated for 30 min in a 37°C CO_2_ incubator. Absorbance was measured at a wavelength of 490 nm using a microplate reader (Molecular Devices, SpectraMax ABS Plus). Cell viability was calculated by subtracting the absorbance of cell-free complete media containing the MTS solution.

### Stress granule quantification

To quantify the number of stress granules in the images, we adjusted the contrast so that the core of 50% of the G3BP1-GFP foci exhibited the maximum fluorescence signal, and we set one-third of the dark-side signals as the minimum fluorescence signals. Next, circular-shaped foci signals with an area of at least 0.25 μm^2^ that were clearly distinguishable from the outer signals were counted as stress granules. Cells showing a half size compared to the average of their size in each image, possibly due to cell division or cell death processes, were excluded from stress granule count analysis.

## Results

### Establishment of colon cells expressing G3BP1-GFP

**Figure 1.**
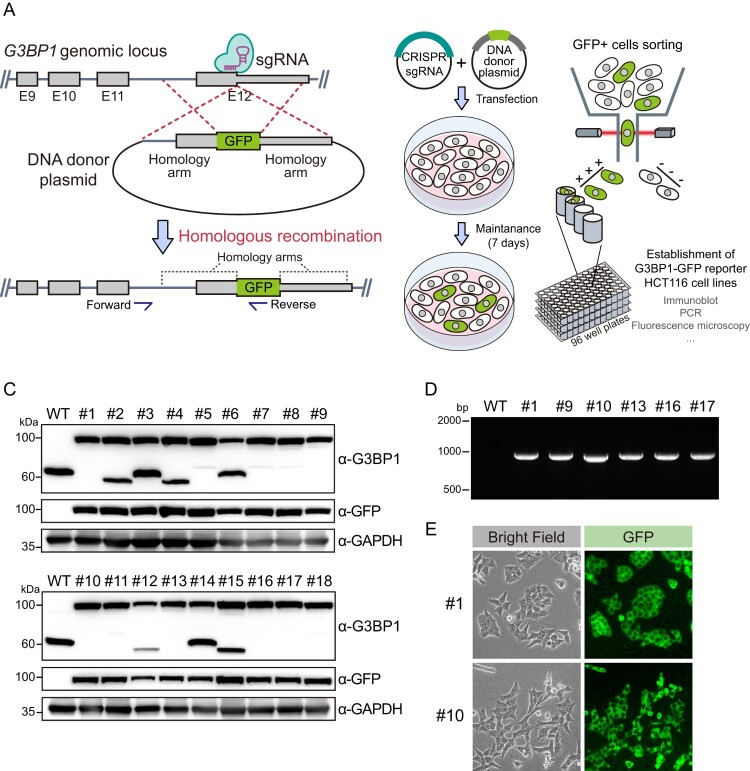
Establishment of HCT116 cells expressing G3BP1-GFP protein. (a) A model representing the process of CRISPR-Cas9-mediated GFP knock-in into the *G3BP1* gene. The boxes and gray lines represent exonic and intronic regions of the *G3BP1* gene, respectively, and the narrow box in exon 12 (E12) shows a 5′ untranslated region. Blue arrows indicate primer binding sites for PCR analysis. (b) Schematic showing procedure for establishing HCT116 cells expressing G3BP1-GFP. Cells showing GFP positive signal were sorted and seeded as one cell per well in culture plates. (c) Immunoblot analysis of G3BP1-GFP protein from each single cell clone. (d) PCR amplification of GFP knock-in *G3BP1* gene. The genomic DNA of each single cell clone was subjected to PCR with primers indicated in (a). (e) Images showing the GFP signal in single clones captured by fluorescence microscopy.

To establish the stress granule reporter cells, we knocked in the *GFP* gene at the *G3BP1* genomic locus using a CRISPR-Cas9-mediated homologous recombination system (Figure 1(a)). Since the NTF-2-like domain in the N-terminus of G3BP1 plays a role in stress granule formation by interacting with PKR, we chose the C-terminus of G3BP1 as the GFP knock-in locus. We cloned a single-guide RNA (sgRNA) and homology arms targeting the C-terminus of G3BP1 CDS into a Cas9 expression plasmid and a plasmid containing GFP, respectively. Both plasmid DNAs were transfected into the human colon cancer cell line, HCT116 (Figure 1(b)). This cell line is generally used for *in vitro* analysis of colon cancer because HCT116 cells can be cultured and proliferated in a commercial medium. Therefore, we planned to establish a stress granule reporter system for this cell line. After transfection, fluorescence-activated cell sorting (FACS) was performed to obtain clones of GFP-positive HCT116 cells. Next, we examined whether the clones expressed G3BP1-GFP. Immunoblot analysis using the G3BP1 antibody showed that the size of G3BP1 protein bands detected from sorted cells was larger (approximately 100 kDa compared to the protein marker) than the size of the G3BP1 protein band (approximately 60 kDa compared to the protein marker) detected in the lysate of wild-type (WT) cells (Figure 1(c)). Furthermore, GFP protein bands in the same protein size region as the upper G3BP1 protein bands were observed only in sorted cells. Therefore, we concluded that HCT116 cells expressing the G3BP1-GFP protein were successfully established. Further PCR analysis in the genomic DNA of these cells using the primers, targeting GFP and upstream genomic DNA region of the 5′ homology arm, validated the GFP knock-in into the *G3BP1* gene in sorted cell lines (Figure 1(d)). Finally, we selected two clones based on cell morphology and confirmed the GFP signal in these cells using fluorescence microscopy (Figure 1(e)). Collectively, we established HCT116 cell lines expressing G3BP1-GFP *via* CRISPR-Cas9-mediated homologous recombination.

### Stress granule formation of G3BP1-GFP

**Figure 2.**
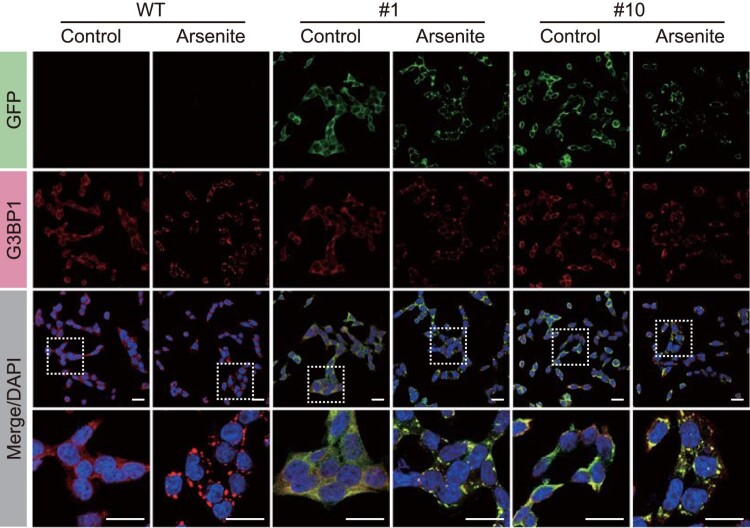
Stress granule formation of G3BP1-GFP protein in HCT116 cells. Immunofluorescence analysis shows GFP (green), G3BP1 (red), and DAPI (blue; nuclei) in WT and G3BP1-GFP expressing HCT116 cells. Scale bars indicate 20 μm.

Next, we confirmed whether the G3BP1-GFP proteins expressed in HCT116 cells were included in the stress granules. Arsenite is a well-known stress granule inducer. Therefore, we treated HCT116 cells expressing G3BP1-GFP with arsenite and performed immunofluorescence analysis using GFP and G3BP1 antibodies (Figure 2). First, we confirmed that arsenite treatment induced the formation of intracellular foci of G3BP1 in WT HCT116 cells. Next, we demonstrated that the GFP signal also forms foci, co-stained with the G3BP1 signal, when arsenite is treated to HCT116 cells expressing G3BP1-GFP. This result revealed that even though GFP was knocked in into the C-terminals of G3BP1 CDS in HCT116 cells, it had no significant impact on stress granule formation, and we concluded that GFP fluorescence signals represent the G3BP1 protein for subsequent experiments.

### Systemic formation of stress granules in colon cells expressing G3BP1-GFP

**Figure 3.**
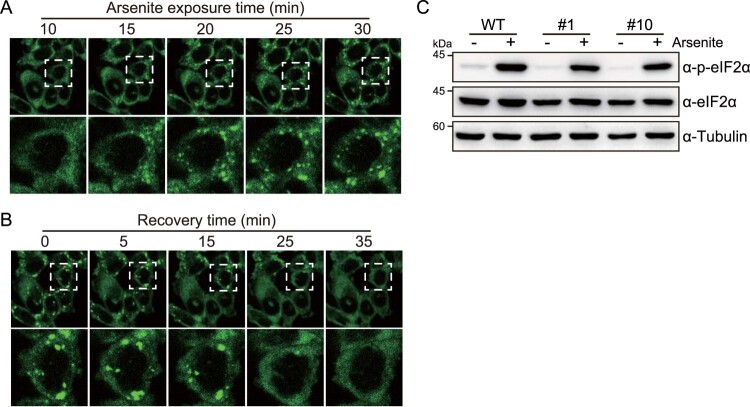
Assessment of stress granule pathway in G3BP1-GFP knock-in HCT116 cells. (a, b) Live image of GFP fluorescence in HCT116 cells expressing G3BP1-GFP. Time series images were captured after 500 μM arsenite treatment for 30 min (a), followed by the removal of the arsenite from the medium (b). (c) Immunoblot analysis showing the phosphorylation of eIF2α by arsenite treatment. WT and G3BP1-GFP expressing HCT116 cells were treated with 500 μM arsenite followed by immunoblot analysis.

To confirm that HCT116 cells express G3BP1-GFP as a stress granule reporter, we tested the systemic and dynamic formation of stress granules. Stress granules are liquid–liquid phase separation-based foci that condense reversibly depending on cellular stress stimuli; thus, stress granules can be rapidly formed by toxic chemicals but can also dissolve rapidly after the removal of toxic chemicals. This prompted us to confirm whether G3BP1-GFP proteins in HCT116 cells could reversibly and rapidly form stress granules upon arsenite treatment. Using confocal microscopy, we observed real-time stress granule formation in HCT116 cells expressing G3BP1-GFP during arsenite treatment in a time series (Figure 3(a)). Moreover, time-series imaging of G3BP1-GFP in HCT116 cells showed dissociation of stress granule foci during the recovery time after removing the arsenite exposure (Figure 3(b)). These results showed that the stress granules containing G3BP1-GFP proteins were rapidly condensed or dissolved, depending on the cellular toxicity in HCT116 cells. Additionally, we evaluated the phosphorylation of eIF2α, the major trigger of stress granule formation, by immunoblot analysis and confirmed that there was no difference in arsenite-induced eIF2α phosphorylation in G3BP1-GFP expressing cells compared with those in WT cells (Figure 3(c)).

**Figure 4.**
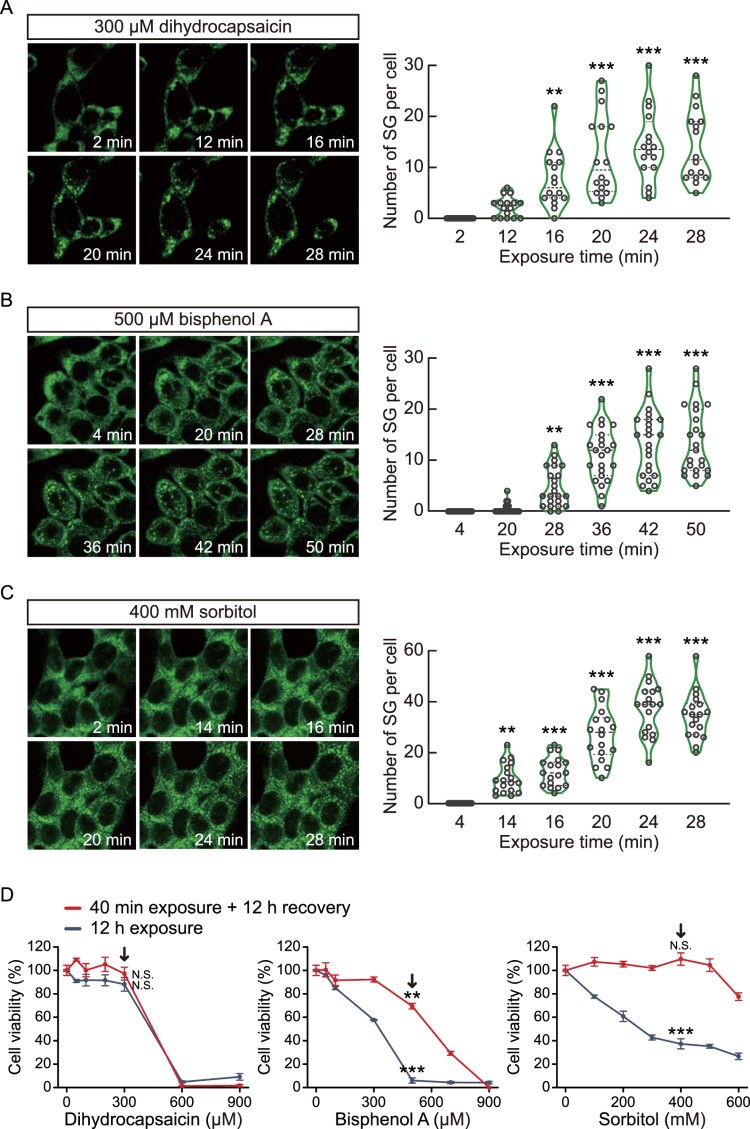
Real-time monitoring of stress granule formation by dihydrocapsaicin, bisphenol A, and sorbitol. (a–c) Time series images (left) of G3BP1-GFP fluorescence of HCT116 cells expressing G3BP1-GFP. Cells were treated with 300 μM dihydrocapsaicin (a), 500 μM bisphenol A (b), or 400 mM sorbitol (c), followed by live imaging under confocal microscopy. Violin plots (right) showing the number of stress granule (SG) per cell at the indicated time point. *p*-values were calculated using one-way ANOVA with Dunnett’s multiple comparison test. **p < 0.01, ***p < 0.001. (d) Cell viability of G3BP1-GFP knock-in HCT116 cells treated with dihydrocapsaicin, bisphenol A, or sorbitol. Cells were treated with chemicals at indicated concentrations for 40 min and then allowed to recover in complete media for 12 h (red lines), or the chemicals were left in the cells for 12 h (blue-gray lines). Cell viability was measured using an MTS assay. Black arrows indicate the concentrations for monitoring stress granule formation. *p*-values were calculated using two-tailed Student’s *t*-test. **p < 0.01, ***p < 0.001. N.S. = not significant.

Next, we determined whether G3BP1-GFP knock-in HCT116 cells could serve as stress granule reporters to assess the toxicity of chemicals in colon cells. To verify this hypothesis, HCT116 cells expressing G3BP1-GFP were treated with chemicals that can repeatedly expose to colon. (1) Dihydrocapsaicin is one of the two major capsaicinoids, along with capsaicin, found in chili peppers, and is the primary activating component for spicy taste; therefore, it is widely used as an additive chemical in industrially manufactured foods (Pena-Alvarez et al. 2009). (2) Bisphenol A is a widely used chemical in the production of plastics, including food containers. Recent studies have identified that bisphenol A is commonly included in industrially manufactured foods and beverages packaged or contained in plastic products (Ballesteros-Gomez et al. 2009; Schecter et al. 2010). (3) Sorbitol, a sugar alcohol, is a natural metabolite produced from the reduction of glucose and can be found in natural foods (Yao et al. 2014). Sorbitol is used as a rectal enema to induce hyperosmolarity in colon tissue, leading to diarrhea. However, the effects of those chemicals on colon cells were not well studied. We treated HCT116 cells expressing G3BP1-GFP protein with 300 μM dihydrocapsaicin, 500 μM bisphenol A, or 400 mM sorbitol, and then monitored the stress granule formation by time series imaging the GFP signals (Figure 4(a–c)). The results revealed that these chemicals significantly increased the number of stress granule foci per cell on HCT116 cells.

Most cytotoxic analyses using *in vitro* cell lines expose the cells to chemicals for more than 12 h. However, numerous molecules are repeatedly exposed to our digestive organs, including the colon, but the exposure time to cells may be short. Therefore, we raised the question of the sensitivity of our real-time stress granule-reporter system for measuring cellular stresses from short-time chemical exposure compared to common cell viability analysis. We treated dihydrocapsaicin, bisphenol A, and sorbitol in HCT116 cells expressing G3BP1-GFP for 40 min or 12 h. Subsequently, cells exposed to chemicals for 40 min were recovered for 12 h in complete media. Next, an MTS assay was performed to analyze the cytotoxicity (Figure 4(d)). From the result, we found that the 40 min exposure to 300 μM dihydrocapsaicin or 400 mM sorbitol showed no significant differences in cell viability assay, even though these concentrations of dihydrocapsaicin and sorbitol induced stress granule formation in less than 20 min of exposure time. On the other hand, the 40 min exposure to 500 μM bisphenol A significantly decreased cell viability. Overall, our results suggest that HCT116 cells expressing G3BP1-GFP can potentially be used for predicting colon cell toxicity of various chemicals by real-time monitoring the stress granule formation.

## Rapid dissociation of bisphenol A induced-stress granules by PERK inhibitor

**Figure 5.**
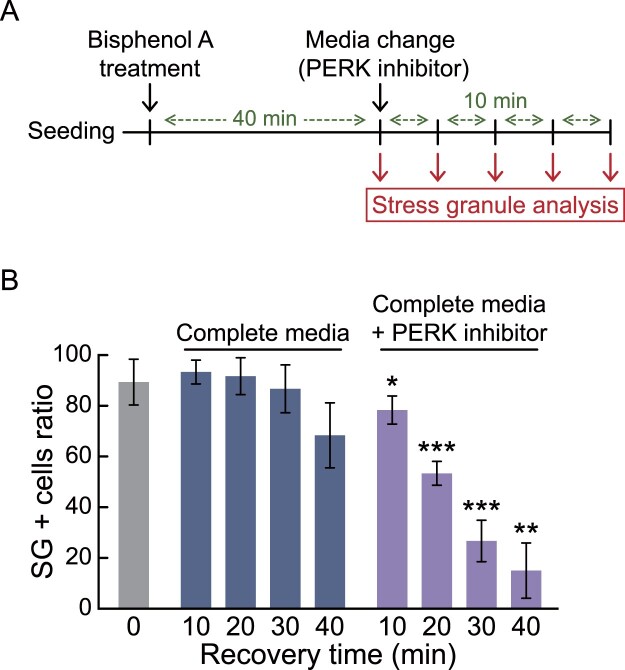
Identification of a PERK inhibitor (GSK2606414) inducing rapid dissociation of bisphenol A-induced stress granules utilizing G3BP1-GFP knock-in HCT116 cells. (a) Schematic showing the procedure for stress granule analysis. (b) Bar graph displaying the ratio of stress granule positive (SG +) cells. G3BP1-GFP knock-in HCT116 cells were treated with 500 μM of bisphenol A for 40 min, followed by changing the media with or without 0.5 μM of PERK inhibitor for 0–40 min. Immunofluorescence analysis using G3BP1 antibody was performed to measure the SG + cell ratio. *p*-values were calculated using two-tailed Student’s *t*-test. *p < 0.05, **p < 0.01, ***p < 0.001 versus cells recovered for the same time in complete media without PERK inhibitor.

Lastly, we next investigate the potential of our established cells to screen the reagents that induces dissociation of stress granules. Taking advantage of our established cell line, which can easily confirm stress granule formation under fluorescence microcopy, we induced stress granules by treating them with 300 μM dihydrocapsaicin, 500 μM bisphenol A, or 400 mM sorbitol for 30 min, and then recovered them with changing the media containing various molecules (Data are not shown), revealed that PERK inhibitor promotes the rapid dissociation of bisphenol A induced-stress granules. To quantify this result, we conducted immunofluorescence analysis in a time-dependent manner (Figure 5(a)). We confirmed that the ratio of stress granule-positive cells was significantly reduced in PERK inhibitor-added media, even for 10 min of treatment time (Figure 5(b)). Overall, we show that G3BP1-GFP knock-in HCT116 cells not only sensitively report the colon cell toxicity of environmental chemicals in short-term exposure but also effectively identify reagents that alleviate the stress granule formation.

## Discussion

In this study, we established the HCT116 cells expressing G3BP1-GFP using CRISPR-Cas9-mediated homologous recombination. We also confirmed that these cells could potentially be used as an *in vitro* colon toxicity monitoring system based on real-time reporting of stress granule formation. Stress granule formation is a highly sensitive and acute defense mechanism that responds to a wide range of cellular stress stimuli, including oxidative stress, ER stress, hypoxia, osmotic stress, unfolded protein responses, and viral infections (Cho et al. 2024; Jung et al. 2023; Kang et al. 2018; Wang et al. 2020; Wang et al. 2021; Zeng et al. 2020). These features may be a powerful advantage in our established cell lines and could contribute to the sensitive reporting of cellular toxicity from a broad range of cellular stresses. We also evaluated the toxicity of dihydrocapsaicin, bisphenol A, or sorbitol by real-time monitoring of stress granule formation. Therefore, conducting a comprehensive screening study of chemicals contained in daily food and orally administered drugs could identify new hazardous substances that have not been previously recognized.

The most common method for assessing chemical toxicity involves measuring cell survival rates after 24 h exposure to chemicals. The half maximal inhibitory concentration (IC_50_) is often used to determine the safe concentration range of chemical exposure. While this approach is favorable for cancer drug development, it has limitations in estimating the toxicity of environmental chemicals that are repeatedly but briefly exposed to cells. Additionally, it is insufficient in revealing the risk of human disease from chronic chemical exposure. In this study, we found that exposure to 300 μM dihydrocapsaicin or 400 mM sorbitol for 40 min in G3BP1-GFP knock-in HCT116 cells induces stress granule formation but does not significantly affect cell viability. This suggests that stress granule formation and cell viability assays may yield different results. Recently, we identified that exposure to Polyhexamethylene guanidine phosphate (PHMG-p) or particulate matter under 10 μm (PM10) in respiratory syncytial virus (RSV) infected human lung cells and human lung organoids induced stress granule formation (Choi, Choi, et al. 2022; Choi, Kim, et al. 2022). Moreover, we revealed that repeated exposure to these chemicals eventually induced DNA damage and cell death. Therefore, we propose that stress granule monitoring systems could serve as an alternative approach for assessing the toxicity of environmental chemicals, reflecting the cellular biological microenvironment more accurately. However, our study only confirmed stress granule formation from exposure to three chemicals. To validate our system as a reliable method for monitoring colon cell toxicity from short-term chemical exposure, further experiments are necessary. These should include High Content Screening (HCS)-based bulk chemical screening assays and validation of disease development risk from chemical exposure in *in vivo* models.

Recently, HCS technology has been developed to automatically image live cells in multi-well culture plates under a microscope and quantitatively analyze cell phenotypes. HCS has been used in compound screening for drug discovery from chemical libraries, genome-wide RNA interference screening to identify genes involved in specific cellular pathways, and toxicological compound testing to identify hazardous chemicals in humans (Bock et al. 2022; Boutros and Ahringer 2008). We speculate that our established G3BP1-GFP reporter cells can be applied to HCS-based experiments not only to evaluate colon toxicity from numerous chemicals easily but also to identify new factors that trigger or inhibit stress granules.

Since not all cytotoxic chemicals trigger stress granule formation, the assessment of stress granule-mediated *in vitro* colonic toxicity may have limited accuracy (Cambronero-Urena et al. 2021). This limitation may also be present in other assessment systems that evaluate cellular toxicity or disease-associated cellular pathways. Nevertheless, our study presented a strategy for the knock-in of GFP into G3BP1 and demonstrated it as a method for establishing a stress granule-reporter cell line. Therefore, our results provide novel insights into the integration of our stress granule reporting system with other systems that report different disease risks, potentially leading to the development of a new colon toxicity evaluation system that can synergistically and accurately report disease risk factors.
